# [Expression of Concern] D-galactose induces astrocytic aging and contributes to astrocytoma progression and chemoresistance via cellular senescence

**DOI:** 10.3892/mmr.2026.14059

**Published:** 2026-11-03

**Authors:** Jingang Hou, Yeejin Yun, Jianjie Xue, Mengqi Sun, Sunchang Kim

Mol Med Rep 20: 4111-4118, 2019; DOI: 10.3892/mmr.2019.10677

Following the publication of the above paper, it was drawn to the Editor's attention by a concerned reader that the p16 antibody that the authors reported to have used for the western blot experiment shown in Fig. 1C (cat. no. ab51243) is specific for the protein known as P16 ARC (or ARPC5), not the apoptosis-associated protein p16INK4a, as was intended.

The authors were contacted by the Editorial Office to offer an explanation for the apparent selection of an unsuitable antibody in this paper, and although they have replied, they have been unable to present the proof of purchase of the antibody in question given the lengthy time period that has elapsed since this study was published. Therefore, owing to the fact that the Editorial Office has been made aware of potential issues surrounding the scientific integrity of this paper, we are issuing an Expression of Concern to notify readers of this potential problem.

